# GC‐MS, alpha‐amylase, and alpha‐glucosidase inhibition and molecular docking analysis of selected phytoconstituents of small wild date palm fruit (*Phoenix pusilla*)

**DOI:** 10.1002/fsn3.3489

**Published:** 2023-06-16

**Authors:** Kumaraswamy Srinivasan, Ammar B. Altemimi, Radhakrishnan Narayanaswamy, Prabhakaran Vasantha Srinivasan, Mazin A. A. Najm, Nasser Mahna

**Affiliations:** ^1^ Department of Biochemistry St. Peter's Institute of Higher Education and Research (SPIHER) Chennai India; ^2^ Department of Food Science, College of Agriculture University of Basrah Basrah Iraq; ^3^ College of Medicine University of Warith Al‐Anbiyaa Karbala Iraq; ^4^ Department of Biochemistry Saveetha Medical College and Hospital, SIMATS (Deemed to be University) Chennai India; ^5^ Department of Bioinformatics, Saveetha School of Engineering SIMATS (Deemed to be University) Chennai India; ^6^ Pharmaceutical Chemistry Department, College of Pharmacy Al‐Ayen University Thi‐Qar Iraq; ^7^ Department of Horticultural Sciences, Faculty of Agriculture University of Tabriz Tabriz Iran

**Keywords:** 1‐formyl‐2,5‐dimethoxy‐6,9,10‐trimethyl‐anthracene, 9,12‐octadecadienoic acid (Z,Z), alpha amylase inhibition, molecular docking, *Phoenix pusilla*

## Abstract

*Phoenix pusilla* (Arecaceae), commonly known as “small wild date palm”, is regarded as one of the underutilized fruit crops in South India. Methanol extract of *P. pusilla* ripened fruits (PPRF) was analyzed for in vitro porcine pancreatic alpha‐amylase (PPAA) and rat small intestine alpha‐glucosidase (RIAG) inhibition activities, and through gas chromatography–mass spectrometry (GC‐MS) analysis. The GC‐MS analysis showed the presence of 25 phytoconstituents from PPRF which was further assessed on the docking behavior of five targeted enzymes diabetes mellitus (DM) namely (i) human aldose reductase, (ii) protein tyrosine phosphatase 1B, (iii) pancreatic alpha‐amylase, (iv) peroxisome proliferator‐activated receptor gamma, and (v) dipeptidyl peptidase IV by using the AutoDock Vina method. In addition to this physicochemical, bioactivity score, absorption, distribution, metabolism, excretion, and toxicity (ADMET) analysis was performed using the Molinspiration and pkCSM free online servers. Methanolic extract of PPRF showed 50% inhibition concentration (IC_50_) at 69.86 and 72.60 μg/mL levels against PPAA and RIAG enzymes activities, respectively. Interestingly in the present study, GC‐MS analysis showed the presence of 25 phytoconstituents from PPRF. Physicochemical analysis of PPRF has exhibited that 13 ligands have complied well with Lipinski's Rule of Five (RoF). With regard to ADMET analysis, one ligand (9,12‐octadecadienoic acid [Z,Z]) has predicated to possess both the hepatotoxicity (HT) and skin sensitization (SS) effect. The docking studies showed that 1‐formyl‐2,5‐dimethoxy‐6,9,10‐trimethyl‐anthracene exhibited the maximum atomic contact energy (ACE) for all the five target enzymes of DM. Thus, the current study suggested that the methanolic extract of PPRF and its phytoconstituents could be considered as potent antidiabetic agents.

## Graphical abstract

The methanolic extract of *Phoenix pusilla* ripened fruits (PPRF) was analyzed through in vitro porcine pancreatic alpha‐amylase (PPAA) inhibition activity assay and gas chromatography–mass‐spectrometry (GC‐MS). The current study suggested that the methanolic extract of PPRF and its phytoconstituents can be considered as potent antidiabetic agents.

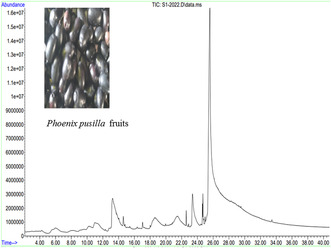

## 1 INTRODUCTION

Plants have been recognized as excellent sources for both diet consumption and medicinal uses in humans. In India, since time immemorial, many plants have been used as a source of medicine (Balamurugan et al., 2019). Several plant parts namely leaves, bark, stem, flowers, roots, and fruits have been known to possess medicinal properties. Herbal drugs have advantages: (i) easily available, (ii) safe, (iii) with minimum side effects, and (iv) affordable when compared with synthetic drugs (Yadav & Singh, 2011). The medicinal values of few plants have been reported to possess chemically active substances that affect the physiological actions of the human body (Pradeep et al., 2014). *Phoenix* belongs to the Arecaceae family, which has approximately 22 species worldwide that include *Phoenix abyssinica*, *P. acaulis*, *P. andamanensis*, *P. atlantica*, *P. caespitose*, *P. canariensis*, *P. canariensis x reclinata*, *P. dactylifera*, *P. farinifera*, *P. leonensis*, *P. loureiroi*, *P. paludosa*, *P. pumila*, *P. pusilla*, *P. reclinata*, *P. reclinata* var. *leonensis*, *P. roebelenii*, *P. rupicola*, *P. spinose*, *P. sylvestris*, *P. theophrasti*, and *P. zeylanica*. Most of *Phoenix* (palm) species are utilized as ornamental plants. Nearly 80% of fruits (dates) from *Phoenix* (palm) species are edible and are regularly consumed in many countries in Asia, Africa, and Europe (Amorós et al., 2014). Leaves of *P. loureiroi* have been used as a natural broom by the tribal communities living in the Nilgiri biosphere reserve (NBR), Western Ghats, India (Rasingam & Jeeva, 2013). Similarly, the fruits (dates) of *P. loureiroi* have been used as folk astringent in intestinal problems (Murugan et al., 2017). In Andhra Pradesh (South India), the fruits (dates) of *P. loureiroi* have been consumed for curing diabetes mellitus (DM) (Pavani et al., 2012). The *Phoenix* (palm) species have been traditionally utilized for treating bronchitis, burning sensation, cough, gastropathy, nephropathy, rheumatism, and sexual debility (Mondal et al., 2021). Similarly, *Phoenix* (palm) species have been reported to possess various biological activities such as analgesic, antidiarrheal, anti‐inflammatory, antimutagenic, antioxidant, and antipyretic (Mondal et al., 2021). *P. loureiroi* (thorn extract) has been reported to possess antimicrobial activity (Jyothsna & Gogu, 2016). Similarly, *Phoenix* (palm) dates/fruits have been reported to possess antibacterial and antifungal activities (Mondal et al., 2021; Perveen et al., 2012). *P. pusilla* fruit has been known to possess aphrodisiac, cardiotonic, carminative, cooling, laxative, and roborant effects. *P. pusilla* fruit has been used for treating burning sensation, cardiac and general debility, consumption, gastropathy, hyperdipsia, fevers, and seminal weakness (Sankar & Shoba, 2017). Thus, the above background engaged us to carry out the current study, where methanol extract of *P. pusilla* ripened fruits (PPRF) were analyzed for in vitro porcine pancreatic alpha‐amylase (PPAA) and rat small intestine alpha‐glucosidase (RIAG) inhibition activities, gas chromatograph–mass spectrometer (GC‐MS) analysis. The GC‐MS analysis showed the presence of 25 phytoconstituents from PPRF was further assessed on the docking behavior of five targeted enzymes of DM namely (i) human aldose reductase (HAR), (ii) protein tyrosine phosphatase 1B (PTP1B), (iii) human pancreatic alpha‐amylase (HPAA), (iv) peroxisome proliferator‐activated receptor gamma (HPPARG), and (v) dipeptidyl peptidase IV (HDPP‐IV) by using the AutoDock Vina method. In addition to these physicochemical properties and bioactivity score, absorption, distribution, metabolism, excretion, and toxicity (ADMET) analysis was performed using the Molinspiration and pkCSM free online servers.

## 2 MATERIALS AND METHODS

Starch (soluble), potassium dihydrogen phosphate, and dipotassium hydrogen phosphate were purchased from Avra Synthesis Private Ltd. Porcine pancreas alpha‐amylase (PPAA), DNS (3,5‐dinitro salicylic acid), acarbose, and methanol were purchased from SRL Private Ltd.

### 2.1 Plant collection and authentication

The PPRF were collected from the Nemili village, near Arakkonam Tamilnadu, India during May and June 2022. Dr. K.N. Sunil Kumar, Research Officer and Head of Pharmacognosy Department, Siddha Central Research Institute (SCRI), Arumbakkam, Chennai has identified and authenticated the plant and fruit. The voucher specimen was prepared and deposited (reference number: P12072202P) in the SCRI herbarium for future reference.

### 2.2 Extraction

The collected PPRF were washed thoroughly in distilled water 3 times, then subjected to sunshade drying after removing seeds, and followed by pulverized into fine powder using a electrical grinder. About 100 g of dry powdered fruits sap (PPRF) were extracted with 150 mL of 70% methanol using the Borosil Soxhlet apparatus for 6–8 h. The selection of methanol for the solvent extraction was considered to deliver many benefits compared to other organic solvents, as it is relatively safer protic solvent with a polarity index value of 5.1 and a dielectric constant of 33.70 at 25°C (Hikmawanti et al., 2021). The 70% ethanol solvent in this research was capable to extract vital phytocompounds from the dry powder of PPRF.

The PPRF extract was then concentrated to dryness in a rotavapor. Half of the portion of the extract was stored in a deep freezer and another half portion of the extract was stored in a desiccator for future applications.

### 2.3 In vitro PPAA inhibition assay

PPAA inhibition assay was carried out by adopting a slightly modified protocol of Shaikh et al. (2017). The soluble starch (0.15%) was used as substrate in 50 mM potassium phosphate buffer (pH 7.0), incubated with 50 μL of the enzyme (PPAA) at 37°C for 30 min. (In the case of test sample tubes, the buffer and sample [PPRF] volume were adjusted in order to maintain the final reaction volume.) Then the biochemical reaction was terminated by the addition of 1 mL of dinitro salicylic acid (DNS) reagent (1% 3,5‐dinitro salicylic acid, 12% sodium potassium tartrate, 0.4 M sodium hydroxide). The test tubes were kept in boiling water for 15 min and then cooled under running tap water. Finally, the absorbance was determined at 540 nm using Epoch 2 (Bio Tek) microplate reader. Acarbose was used as a positive control. All the experiments were performed in triplicate. The data were fitted on a polynomial (regression) model, whereas vertical bars indicate standard error (±SEM), and Microcal Software (Sigma plot 11) was used to plot the graph.

### 2.4 In vitro RIAG inhibition assay

RIAG preparation and as well inhibition assay was carried out by adopting Sakayanathan et al. (2018) protocol. The maltose (1 mM) was used as substrate in 100 mM potassium phosphate buffer (pH 7.0), incubated with an aliquot of the enzyme (RIAG) at 37°C for 30 min. (In the case of test sample tubes, the buffer and sample [RIAG] volume were adjusted in order to maintain the final reaction volume.) After incubation, the concentration of glucose was measured by using a glucose estimation kit as per manufacturer's instruction (Coral Clinical Systems). Finally, the absorbance was determined at 505 nm using Epoch 2 (Bio Tek) microplate reader. Acarbose was used as a positive control. All the experiments were performed in triplicate. The data were fitted on a polynomial (regression) model, whereas vertical bars indicate standard error (±SEM) and Microcal Software (Sigma plot 11) was used to plot the graph.

### 2.5 GC‐MS analysis of PPRF

One microliter of PPRF sample was taken for GC‐MS (Agilent Technology, 7890B Model equipped with 5977A Mass Detector) analysis to determine the chemical constituents present in the PPRF sample. GC‐MS was performed using the following conditions (i) column: C18 Silica (normal phase), (ii) oven temperature: 60°C, (iii) injection temperature: 250°C, and (iv) helium was used as carrier gas at the split ratio of (1:1). Finally, the obtained mass spectrum was interpreted and compared with the National Institute of Standards and Technology (NIST) library database (Srikalyani & Ilango, 2020).

### 2.6 Ligand preparation

Chemical structures of 25 ligands (*P. pusilla* fruit constituents) namely (i) 6‐oxabicyclo [3,1,0] hexan‐2‐one (CID 242082), (ii) 4H‐pyran‐4‐one, 2,3‐dihydro‐3,5‐dihydroxy‐6‐methyl (CID 119838), (iii) 1‐butoxypropan‐2‐yl 2‐methylbutanoate (CID 91693251), (iv) glycerin (CID 753), (v) dimethylamine, *N*‐(neopentyloxy) (CID 548341), (vi) tricyclo [2,2,1,0 (2,6)] heptane, 1,3,3‐trimethyl (CID 79022), (vii) benzene, 1‐ethynyl‐4‐fluoro (CID 11171265), (viii) 5‐hydroxymethylfurfural (CID 237332), (ix) phenol, 2‐methoxy‐3‐(2‐propenyl) (CID 596373), (x) cetene (CID 12395), (xi) 7‐epi‐*trans*‐sesquisabinene hydrate (CID 100930863), (xii) Z‐10‐pentadecen‐1‐ol (CID 5364483), (xiii) dodecanoic acid, methyl ester (CID 8139), (xiv) trichloroacetic acid, 4‐tetradecyl ester (CID 534423), (xv) decanoic acid, 3‐methyl (CID 143696), (xvi) methyl tetradecanoate (CID 31284), (xvii) 3‐deoxy‐d‐mannoic lactone (CID 541561), (xviii) hexadecanoic acid, methyl ester (CID 8181), (xix) benzenepropanoic acid, 3,5‐*bis*(1,1‐dimethylethyl)‐4‐hydroxy‐, methyl ester (CID 62603), (xx) *n*‐hexadecanoic acid (CID 985), (xxi) 1‐formyl‐2,5‐dimethoxy‐6,9,10‐trimethyl‐anthracene (CID 626348), (xxii) 9‐octadecenoic acid, methyl ester (CID 5280590), (xxiii) methyl stearate (CID 8201), and (xxiv) 9,12‐octadecadienoic acid (Z,Z) were downloaded from PubMed. One more unavailable structure of ligand (β‐allyloxypropionic acid) and the selected 24 ligands were drawn by using ChemBio Draw Ultra 12.0 and the molecular mechanics (MM2) energy minimization of ligands was performed by ChemBio 3D Ultra 12.0, according to the reported protocol (Vijayakumar et al., 2017). Furthermore, the prepared ligands were converted into the pdbqt file format using the Open Babel free software.

### 2.7 Physicochemical property and bioactive score analysis

The Molinspiration free online server was used to analyze physicochemical property and bioactive score of 25 selected ligands of *P. pusilla* (Mohan et al., 2022).

### 2.8 ADMET analysis

The pkCSM online server was used to analyze the ADMET properties of 25 selected ligands of PPRF (Pires et al., 2015).

### 2.9 Target protein identification and preparation

The three–dimensional (3D) structure of the target proteins: (i) human aldose reductase (PDB ID: IUS0 with resolution of 0.66 A°), (ii) protein tyrosine phosphatase 1B (PDB ID: 2QBS with a resolution of 2.10 A°), (iii) human pancreatic alpha‐amylase (PDB ID: 2QV4 with a resolution of 1.97 A°), (iv) human peroxisome proliferator‐activated receptor gamma (PDB ID: 3CS8 with a resolution of 2.30 A°), and (v) human dipeptidyl peptidase IV (PDB ID: 4A5S with a resolution of 1.62 A°) were obtained from the Research Collaboratory for Structural Bioinformatics (RCSB) Protein Data Bank (www.rcsb.org). A chain of these five proteins was preprocessed by removing other chains (B and C) and ligands, in addition to the crystallographically observed water particles (water without hydrogen bonds). UCSF Chimera software (www.cgi.ucsf.edu/chimera) was utilized to prepare the abovementioned protein (Radhakrishnan, 2017). Then each prepared protein was opened in Autodock 1.5.6 (MGL) tool, then polar hydrogen atoms and Kollman charges were added to each protein, and finally, protein (macromolecule) was saved the pdbqt file format for further docking analysis.

#### 2.9.1 Active site prediction

The active site of each protein was determined using the active site prediction web server (http://www.scfbio‐iitd.res.in/dock/ActiveSite.jsp). The pre‐registered email id was provided; the server will provide the active sites of each target protein. The active site of (i) human aldose reductase (*x* = 13.294, *y* = −12.810, *z* = 15.508), (ii) protein tyrosine phosphatase 1B (*x* = 46.950, *y* = 16.950, *z* = 30.572), (iii) human pancreatic alpha‐amylase (*x* = 20.876, *y* = 50.513, *z* = 28.753), (iv) human peroxisome proliferator‐activated receptor gamma (*x* = 16.659, *y* = −1.370, *z* = 31.043), and (v) human dipeptidyl peptidase‐IV (*x* = 8.178, *y* = 24.024, *z* = 55.181).

#### 2.9.2 Molecular docking studies

The molecular docking studies were carryout by using the AutoDock Vina in Linux command mode (Vázquez‐Jiménez et al., 2023). Initially, a grid box was set according to the active site of the crystal structure of each protein mentioned in the above active site prediction section. The first‐ranked pose for each ligand was considered for the binding energy calculation and similarly, binding sites were analyzed by using the protein–ligand interaction profiler (PLIP) free web server.

## 3 RESULTS AND DISCUSSION

**FIGURE 1:**
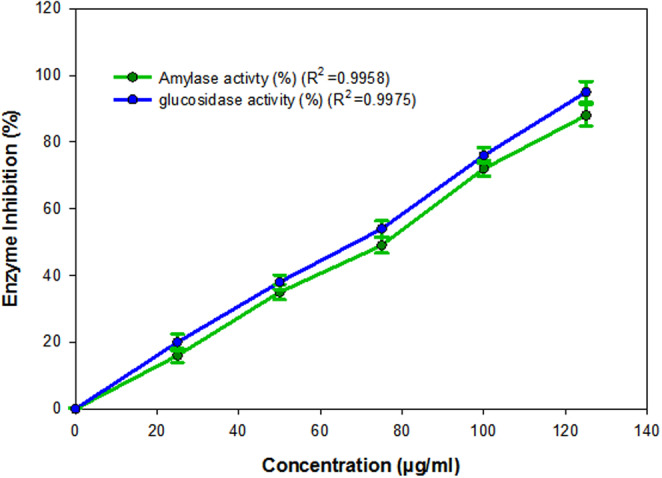
The bar diagram of methanolic extract of *Phoenix pusilla* ripened fruit (PPRF) against porcine pancreatic alpha‐amylase (PPAA) and alpha‐glucosidase inhibition. The data were fitted on a polynomial (regression) model, whereas vertical bars indicate standard error (±SEM) and Microcal Software (Sigma plot 11) was used to plot the graph.

*Phoenix pusilla* (Arecaceae) is commonly known as “Parusakah” (in Sanskrit) and “Siruinju” (in Tamil). *P. pusilla* has been listed as one of the top 250 ayurvedic medicinal plants in India (Saravanan et al., 2021). The different parts of *P. pusilla* such as leaves, fruits, barks, and roots have been well documented (Charaka and Sushruta) and prescribed by Ayurvedic practitioners. Moreover, “sharbat‐e‐phaalsaa” (Unani squash), a summer refreshing drink/juice prepared using *P. pusilla* fruits, has been used as an appetizer and cardiac tonic in India (Roop, 2018). Furthermore, *P. pusilla* unripened fruits have been known to possess biological activities such as antidiabetic and antioxidant (Sankar & Shoba, 2017). Thus, the abovementioned background engaged us to carry out the present study using the PPRF (underutilized fruit) of South India. The organoleptic feature of PPRF has been reported earlier and displays the following characteristics: (i) orange‐red color, (ii) dates flavor odor, (iii) sweet taste, and (iv) oval shape (Farhanaz et al., 2017). About 10.01 ± 0.60 g of methanolic extract was obtained from 100 g of PPRF dry powdered extraction, which approximately accounts for 10% yield. In the current in vitro study, the inhibition potential of the methanolic extract of PPRF was determined against PPAA. Methanolic extract of PPRF showed a dose‐dependent manner inhibition activity against PPAA (Figure 1). The 50% inhibition concentration (IC_50_) of methanolic extract of PPRF was found to be 72.60 ± 0.7 μg/mL, whereas for the control (acarbose) it was found to be 26.31 ± 0.3 μg/mL, respectively. Similarly, the methanolic extract of PPRF showed a dose‐dependent manner inhibition activity against RIAG. The IC_50_ of methanolic extract of PPRF was found to be 69.86 ± 0.9 μg/mL, whereas for the control (acarbose) it was found to be 15.34 ± 0.4 μg/mL, respectively.

**FIGURE 2:**
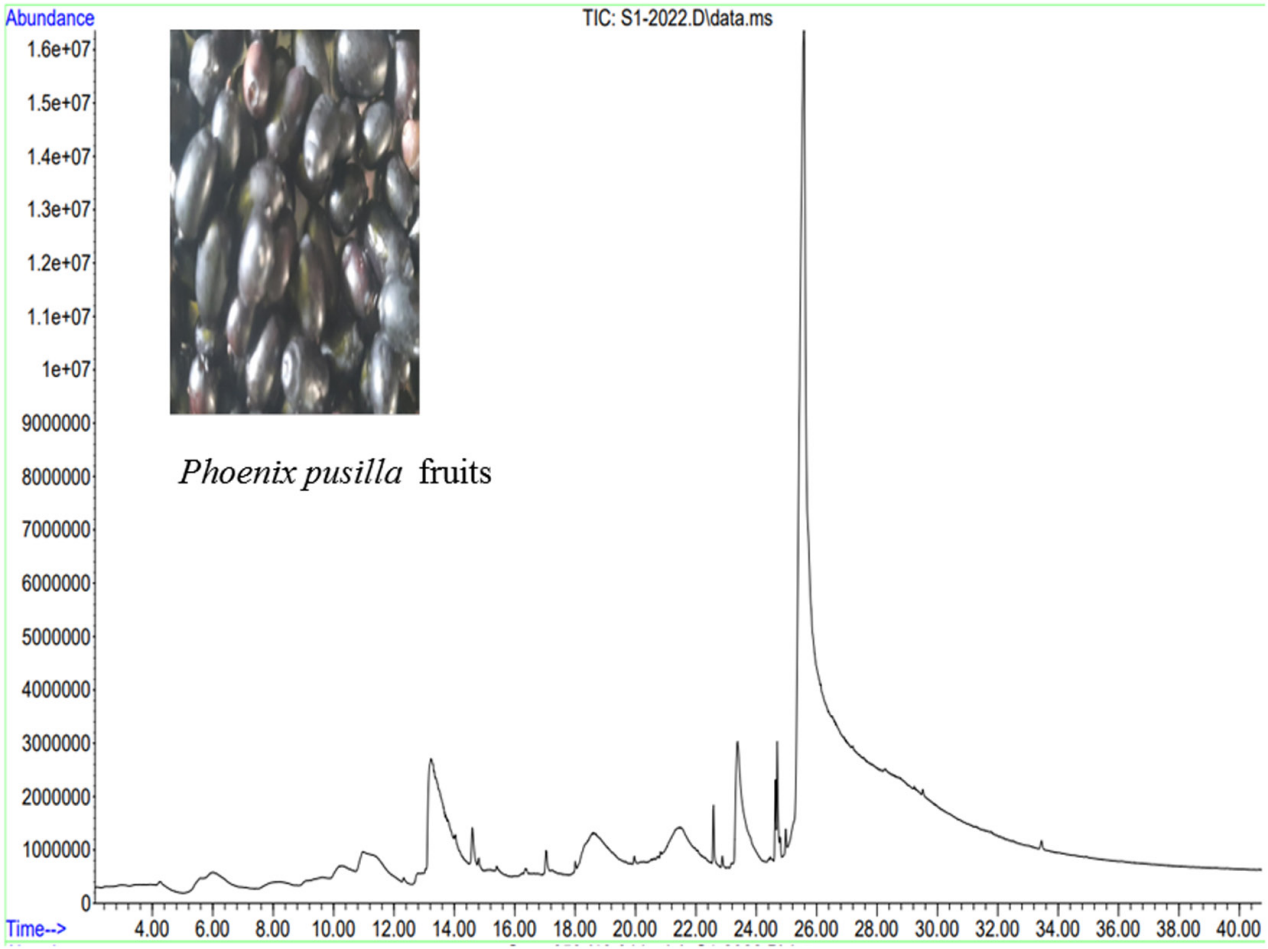
The GC‐MS chromatogram of the methanolic extract of *Phoenix pusilla* ripened fruit (PPRF). *Note*: *x*‐axis: time (minutes); *y*‐axis: abundance (arbitrary units).

GC‐MS chromatogram of the methanolic extract of PPRF revealed the presence of 25 peaks, which suggests the existence of 25 phytochemicals (Figure 2). The phytoconstituents were identified in the methanolic extract of PPRF (Tables 1 and 2). Interestingly in the present study, GC‐MS analysis showed the presence of phytoconstituents namely (i) 9,12‐octadecadienoic acid (Z,Z) (54.720%), (ii) 5‐hydroxymethylfurfural (10.733%), (iii) *n*‐hexadecanoic acid (6.774%), (iv) decanoic acid, 3‐methyl (6.447%), (v) 3‐deoxy‐d‐mannoic lactone (4.758%), (vi) dimethylamine, *N*‐(neopentyloxy) (4.734%), (vii) 4H‐pyran‐4‐one, 2,3‐dihydro‐3,5‐dihydroxy‐6‐methyl (2.376%), (viii) glycerin (1.61%), and (ix) beta‐allyloxypropionic acid (1.595%). To the best of our knowledge, we are the first to demonstrate the PPAA inhibition activity as well as GC‐MS analysis of PPRF, though the unripened fruit inhibition activity has been reported previously by Sankar and Shoba (2017).

**TABLE 1:** The GC‐MS profiling of the methanolic extract of *Phoenix pusilla* ripened fruit (PPRF).

Peak number	RT	Relative abundance (%)	Phytoconstitutent
1	5.702	0.821	6‐Oxabicyclo[3.1.0]hexan‐2‐one
2	5.994	2.376	4H‐Pyran‐4‐one, 2,3‐dihydro‐3,5‐dihydroxy‐6‐methyl
3	8.218	1.595	β‐Allyloxypropionic acid
4	9.662	0.870	1‐Butoxypropan‐2‐yl 2‐methylbutanoate
5	10.298	1.611	Glycerin
6	10.960	4.734	Dimethylamine, *N*‐(neopentyloxy)
7	12.324	0.119	Tricyclo[2.2.1.0(2,6)]heptane, 1,3,3‐trimethyl
8	13.052	0.783	Benzene, 1‐ethynyl‐4‐fluoro
9	13.224	10.668	5‐Hydroxymethylfurfural
	14.032	0.065	
10	14.588	0.421	Phenol, 2‐methoxy‐3‐(2‐propenyl)‐
11	14.800	0.047	Cetene
12	15.410	0.063	7‐Epi‐*trans*‐sesquisabinene hydrate
13	16.350	0.075	Z‐10‐Pentadecen‐1‐ol
14	17.038	0.343	Dodecanoic acid, methyl ester
15	18.005	0.127	Trichloroacetic acid, 4‐tetradecyl ester
16	18.601	6.447	Decanoic acid, 3‐methyl
17	19.965	0.135	Methyl tetradecanoate
18	21.435	4.758	3‐Deoxy‐d‐mannoic lactone
19	22.587	0.619	Hexadecanoic acid, methyl ester
20	22.879	0.118	Benzenepropanoic acid, 3,5‐*bis*(1,1‐dimethylethyl)‐4‐hydroxy‐, methyl ester
21	23.382	6.774	*n*‐Hexadecanoic acid
22	24.640	0.661	1‐Formyl‐2,5‐dimethoxy‐6,9,10‐trimethyl‐anthracene
23	24.693	0.780	9‐Octadecenoic acid, methyl ester
24	24.971	0.273	Methyl stearate
25	25.580	54.720	9,12‐Octadecadienoic acid (Z,Z)‐

Abbreviation: RT, retention time.

**TABLE 2:** The phytoconstitutents identified from the methanolic extract of *Phoenix pusilla* ripened fruit (PPRF) by GC‐MS analysis.

S. no.	Name of phytochemical	IUPAC name	Previously reported biological activity	Reference
1.	6‐Oxabicyclo (3,1,0) hexan‐2‐one	6‐Oxabicyclo[3.1.0]hexan‐2‐one	Not reported	Not available
2.	4H‐Pyran‐4‐one, 2,3‐dihydro‐3,5‐dihydroxy‐6‐methyl	3,5‐Dihydroxy‐6‐methyl‐2,3‐dihydropyran‐4‐one	Antioxidant activity	Dilek Tepe and Doyuk (2020)
3.	Beta‐allyloxypropionic acid	3‐Prop‐2‐enoxypropanoic acid	Antiarthritic activity	Jin et al. (2019)
4.	1‐Butoxypropan‐2‐yl 2‐methylbutanoate	1‐Butoxypropan‐2‐yl 2‐methylbutanoate	Not reported	Not available
5.	Glycerin	Propane‐1,2,3‐triol	Not reported	Not available
6.	Dimethylamine, *N*‐(neopentyloxy)	*N*‐(2,2‐dimethylpropoxy)‐*N*‐methylmethanamine	Hepatoprotective activity	Ahmed et al. (2019)
7.	Tricyclo [2,2,1,0(2,6)]heptane, 1,3,3‐trimethyl	1,3,3‐Trimethyltricyclo[2.2.1.0(2,6)]heptane	Antifungal activity	Hussein et al. (2020)
8.	Benzene,1‐ethynyl‐4‐fluoro	1‐Ethynyl‐4‐fluoro‐2‐(2‐methylprop‐1‐enyl)benzene	Antioxidant activity	Sunday et al. (2021)
9.	5‐Hydroxymethylfurfural	5‐(Hydroxymethyl)furan‐2‐carbaldehyde	Anticancer activity	Joel and Maharjan (2021)
10.	Phenol, 2‐methyoxy‐3‐(2‐propenyl)	2‐Methoxy‐3‐prop‐2‐enylphenol	Antibacterial activity	Shareef et al. (2016)
11.	Cetene	Hexadec‐1‐ene	Antioxidant activity	Subramanian et al. (2020)
12.	7‐Epi‐*trans*‐sesquisabinene hydrate	(1*S*,2*S*,5*R*)‐2‐Methyl‐5‐[(2*S*)‐6‐methylhept‐5‐en‐2‐yl]bicyclo[3.1.0]hexan‐2‐ol	Antimicrobial activity	Kumar et al. (2021)
13.	Z‐10‐Pentadecen‐1‐ol	(*Z*)‐Pentadec‐10‐en‐1‐ol	Antibacterial activity	Akilandeswari et al. (2015)
14.	Dodecanoic acid, methylester	Methyl dodecanoate	Antibacterial activity	Shaheed et al. (2019)
15.	Trichloroacetic acid, 4‐tetradecyl ester	Tetradecan‐4‐yl 2,2,2‐trichloroacetate	Antioxidant and cytotoxicity activities	Bourhia et al. (2020)
16.	Decanoic acid, 3‐methyl	3‐Methyldecanoic acid	Not reported	Not available
17.	Methyl tetradecanoate	Methyl tetradecanoate	Anticancer activity	Ukwubile et al. (2019)
18.	3‐Deoxy‐d‐mannoic lactone	3,5‐Dihydroxy‐6‐(hydroxymethyl)oxan‐2‐one	Antioxidant and alpha‐glucosidase inhibitor activities	Van Chen et al. (2022)
19.	Hexadecanoic acid, methyl ester	Methyl hexadecanoate	Anticancer activity	Ukwubile et al. (2019)
20.	Benzenepropanoic acid, 3,5‐*bis*(1,1‐dimethylethyl)‐4‐hydroxy‐, methyl ester	Methyl 3‐(3,5‐di*tert*‐butyl‐4‐hydroxyphenyl)propionate	Antibacterial activity	Alqahtani et al. (2020)
21.	*n*‐Hexadecanoic acid	Hexadecanoic acid	Antidiabetic activity	Renganathan et al. (2021)
22.	1‐Formyl‐2,5‐dimethoxy‐6,9,10‐trimethyl‐anthracene	2,5‐Dimethoxy‐6,9,10‐trimethylanthracene‐1‐carbaldehyde	Not reported	Not available
23.	9‐Octadecenoic acid, methyl ester	Methyl (*E*)‐octadec‐9‐enoate	Antiandrogenic, anticancer, anti‐inflammatory, hypocholesterolemic and 5‐alpha reductase inhibitor activities	Krishnamoorthy and Subramaniam (2014)
24.	Methyl stearate	Methyl octadecanoate	Anticancer activity	Ukwubile et al. (2019)
25.	9,12‐Octadecadienoic acid (Z,Z)‐	(9*Z*,12*Z*)‐Octadeca‐9,12‐dienoic acid	Antidiabetic activity	Sharma et al. (2021)

In the current study, physicochemical analysis of PPRF has exhibited that 13 ligands have complied well with Lipinski's Rule of Five (RoF), whereas 12 ligands have exhibited one violation (Table 3).

**TABLE 3:** Physicochemical analysis of 25 selected (*Phoenix pusilla*) ligands using Molinspiration free web server.

Ligand number	Log*P* ^a^	natoms^b^	MW^c^	nON^d^	nOHNH^e^	nviolations^f^	nrotb^g^
1	−0.29	7	98.10	2	0	0	0
2	−0.46	10	144.13	4	2	0	0
3	−3.08	9	131.11	4	0	0	5
4	3.58	15	216.32	3	0	0	9
5	−1.60	6	92.09	3	3	0	2
6	2.00	9	131.22	2	0	0	3
7	3.40	10	136.24	0	0	0	0
8	3.50	13	174.22	0	0	0	1
9	0.56	9	126.11	3	1	0	2
10	2.27	12	164.20	2	1	0	3
11	8.17	16	224.43	0	0	1	13
12	4.40	16	222.37	1	1	0	4
13	6.19	16	226.40	1	1	1	12
14	5.35	15	214.35	2	0	1	11
15	8.35	21	359.76	2	0	1	14
16	4.03	13	186.29	2	1	0	8
17	6.36	17	242.40	2	0	1	13
18	−1.63	11	162.14	5	3	0	1
19	7.37	19	270.46	2	0	1	15
20	5.24	21	292.42	3	1	1	6
21	7.06	18	256.43	2	1	1	14
22	5.22	23	308.38	3	0	1	3
23	7.89	21	296.50	2	0	1	16
24	8.32	21	298.51	2	0	1	17
25	6.86	20	280.45	2	1	1	14

^a^ Octanol–water partition coefficient.

^b^ The Number of nonhydrogen atoms.

^c^ Molecular weight.

^d^ The Number of hydrogen bond acceptors [O and N atoms].

^e^ The Number of hydrogen bond donors [OH and NH groups].

^f^ The Number of Lipinski's Rule of Five violations.

^g^ The Number of rotatable bonds.

With regard to the bioactivity score analysis of PPRF, only 9,12‐octadecadienoic acid (Z,Z) (one ligand) has shown an active (>0) bioactivity score against all the descriptors except kinase inhibitor (Table 4).

**TABLE 4:** Bioactivity score analysis of 25 selected (*Phoenix pusilla*) ligands using Molinspiration free web server.

Ligand number	G protein coupled receptors ligand	Ion channel modulator	Kinase inhibitor	Nuclear receptor ligand	Protease inhibitor	Enzyme inhibitor
1	−3.38	−2.83	−3.55	−2.99	−2.85	−2.71
2	−1.59	−0.96	−2.25	−1.60	−1.53	−0.65
3	−2.27	−1.54	−2.37	−2.30	−1.86	−1.48
4	−0.47	−0.28	−1.00	−0.50	−0.45	0.01
5	−3.37	−3.37	−3.54	−3.48	−3.52	−3.17
6	−1.78	−1.16	−2.35	−2.10	−2.12	−1.24
7	−0.81	−0.34	−1.36	−1.05	−0.94	−0.58
8	−0.45	0.18	−0.18	−0.36	−0.73	0.10
9	−2.71	−2.19	−2.92	−2.73	−3.21	−2.24
10	−0.84	−0.35	−1.20	−0.74	−1.32	−0.44
11	−0.29	−0.03	−0.55	−0.22	−0.39	−0.04
12	−0.12	0.12	−0.63	0.31	−0.04	0.22
13	−0.16	0.02	−0.38	−0.15	−0.31	0.12
14	−0.41	−0.13	−0.73	−0.43	−0.46	−0.11
15	−0.11	−0.28	−0.38	−0.24	−0.28	−0.18
16	−0.35	−0.05	−0.97	−0.34	−0.36	−0.05
17	−0.24	−0.07	−0.51	−0.24	−0.28	−0.02
18	−0.53	−0.19	−1.12	−0.34	−0.35	0.35
19	−0.11	−0.05	−0.34	−0.09	−0.13	0.04
20	0.07	0.09	−0.22	0.35	−0.04	0.15
21	0.02	0.06	−0.33	0.08	−0.04	0.18
22	−0.11	−0.18	−0.11	−0.07	−0.36	0.04
23	0.03	−0.03	−0.25	0.06	−0.02	0.12
24	−0.03	−0.04	0.23	0.00	−0.03	0.05
25	0.29	0.17	−0.16	0.31	0.12	0.38

The ADMET analysis of PPRF, where one ligand [9,12‐octadecadienoic acid (Z,Z)] has predicated to possess both the hepatotoxicity (HT) and skin sensitization (SS) effect (Table 5). The physicochemical properties, bioactivity, and ADMET have been reported as pre‐required properties to know before performing the docking studies (Mohan et al., 2022).

**TABLE 5:** Absorption, distribution, metabolism, excretion, and toxicity (ADMET) analysis of 25 selected (*Phoenix pusilla*) ligands using pkCSM free online server.

Ligand number	A	D	M							E	T	
	GI	BBB	CYP 2D6	CYP 3A4	CYP A12	CYP 2C19	CYP 2C9	CYP 2D6	CYP 3A4	TC	HT	SS
1	100	−0.63	No	No	No	No	No	No	No	0.42	No	Yes
2	83.5	−0.3	No	No	No	No	No	No	No	0.50	No	No
3	98.6	−0.35	No	No	No	No	No	No	No	0.86	No	No
4	96.3	0	No	No	No	No	No	No	No	1.67	No	Yes
5	74.2	−0.36	No	No	No	No	No	No	No	0.72	No	No
6	96.7	0.15	No	No	No	No	No	No	No	0.42	No	Yes
7	93.9	0.85	No	Yes	No	No	No	No	No	−0.1	No	No
8	95.6	0.52	No	No	Yes	No	No	No	No	0.25	No	Yes
9	95.8	−0.36	No	No	No	No	No	No	No	0.61	No	No
10	93.2	0.41	No	No	Yes	No	No	No	No	0.37	No	Yes
11	91.5	0.95	No	Yes	Yes	No	No	No	No	1.95	No	Yes
12	93.7	0.66	No	No	No	Yes	No	No	No	1.11	No	Yes
13	90.6	0.77	No	Yes	Yes	No	No	No	No	1.88	No	Yes
14	93.7	0.67	No	No	No	No	No	No	No	1.72	No	Yes
15	90.2	0.60	No	Yes	No	No	No	No	No	0.50	No	Yes
16	93.2	0.15	No	No	No	No	No	No	No	1.51	No	Yes
17	93.0	0.71	No	Yes	Yes	No	No	No	No	1.79	No	Yes
18	65.9	−0.43	No	No	No	No	No	No	No	0.67	No	No
19	92.3	0.75	No	Yes	Yes	No	No	No	No	1.86	No	Yes
20	94.1	−0.45	No	Yes	Yes	Yes	Yes	No	Yes	0.87	No	Yes
21	92.0	−0.11	No	Yes	No	No	No	No	No	1.76	No	Yes
22	96.1	0.19	No	Yes	Yes	Yes	Yes	No	No	0.21	No	No
23	92.1	0.78	No	Yes	Yes	No	No	No	No	1.98	No	Yes
24	91.6	0.79	No	Yes	Yes	No	No	No	No	1.93	No	Yes
25	92.3	−0.14	No	Yes	Yes	No	No	No	No	1.94	Yes	Yes

Abbreviations: 1A2, cytochrome P450 1A2 inhibitor; 2C19, cytochrome P450 2C19 inhibitor; 2C9, cytochrome P450 2C9 inhibitor; 2D6, cytochrome P450 2D6 substrate; 2D6, cytochrome P450 2D6 inhibitor; 3A4, cytochrome P450 3A4 substrate; 3A4, cytochrome P450 3A4 inhibitor; A, absorption; BBB, blood–brain barrier permeant; D, distribution; E, excretion; GI, gastrointestinal; HT, hepatotoxicity; M, metabolism; SS, skin sensitization; T, toxicity; TC, total clearance (log mL/min/kg).

**FIGURE 3:**
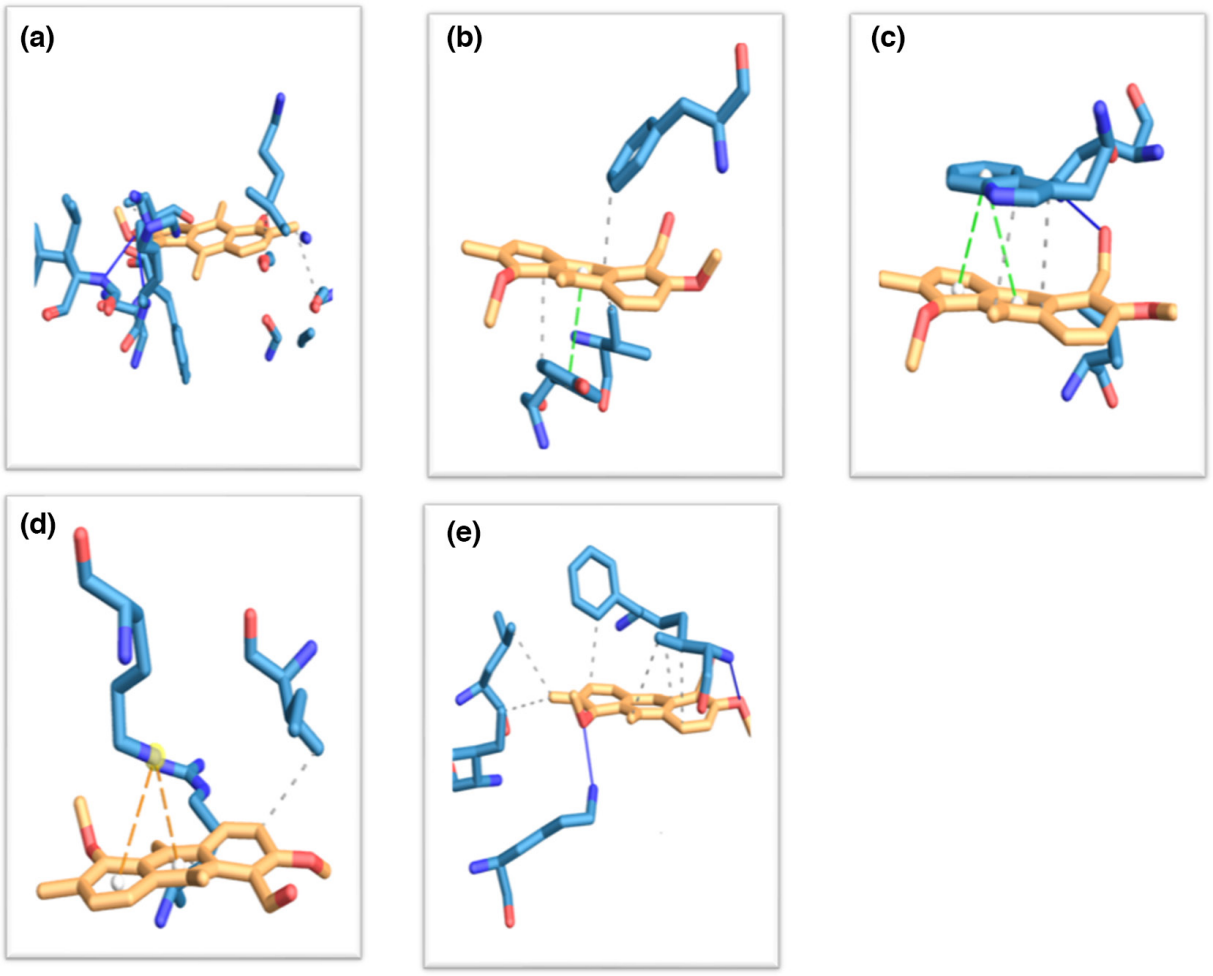
The interaction analysis of ligand 22 (1‐formyl‐2,5‐dimethoxy‐6,9,10‐trimethyl‐anthracene) with that of (a) HAR – human aldose reductase, (b) PTP1B – human protein tyrosine phosphatase 1B, (c) HPAA – human pancreatic alpha‐amylase, (d) HPPARG – human peroxisome proliferator‐activated receptor gamma, and (e) HDPP‐IV^v^ – human dipeptidyl peptidase IV using the protein–ligand interaction profiler (PLIP). *Note*: Dotted lines represent the hydrogen bond between the ligand 22 (1‐formyl‐2,5‐dimethoxy‐6,9,10‐trimethyl‐anthracene) with the five targeted enzymes namely (a) HAR – human aldose reductase, (b) PTP1B – human protein tyrosine phosphatase 1B, (c) HPAA – human pancreatic alpha‐amylase, (d) HPPARG – human peroxisome proliferator‐activated receptor gamma, and (e) HDPP‐IV^v^ – human dipeptidyl peptidase IV.

The docking studies showed that 1‐formyl‐2,5‐dimethoxy‐6,9,10‐trimethyl‐anthracene had exhibited the maximum atomic contact energy (ACE) of −10.7 (kcal/mol) with human aldose reductase (HAR) (Table 6). The binding energy results showed the following (highest to lowest) order: 1‐formyl‐2,5‐dimethoxy‐6,9,10‐trimethyl‐anthracene (−10.7 kcal/mol), <7‐epi‐*trans*‐sesquisabinene hydrate (−10.0 kcal/mol), <benzene propanoic acid, 3,5‐*bis*(1,1‐dimethylethyl)‐4‐hydroxy‐, methyl ester (−9.5 kcal/mol), <benzene, 1‐ethynyl‐4‐fluoro (−8.9 kcal/mol), <trichloroacetic acid, 4‐tetradecyl ester (−8.4 kcal/mol), <phenol, 2‐methoxy‐3‐(2‐propenyl) and 9,12‐octadecadienoic acid (Z,Z) (−8.2 kcal/mol), <hexadecanoic acid, methyl ester (−8.1 kcal/mol), <Z‐10‐pentadecen‐1‐ol, methyl tetradecanoate and 9‐octadecenoic acid, methyl ester (−8.0 kcal/mol), <tricyclo[2.2.1.0(2,6)]heptane, 1,3,3‐trimethyl and decanoic acid, 3‐methyl (−7.8 kcal/mol), <*n*‐hexadecanoic acid (−7.7 kcal/mol), <cetene and methyl stearate (−7.6 kcal/mol), <1‐butoxypropan‐2‐yl 2‐methylbutanoate and 3‐deoxy‐d‐mannoic lactone (−7.5 kcal/mol), <4H‐pyran‐4‐one, 2,3‐dihydro‐3,5‐dihydroxy‐6‐methyl (−7.2 kcal/mol), <5‐hydroxymethylfurfural (−6.8 kcal/mol), <dimethylamine, *N*‐(neopentyloxy) (−6.1 kcal/mol), <β‐allyloxypropionic acid (−6.0 kcal/mol), <6‐oxabicyclo[3.1.0] hexan‐2‐one (−5.7 kcal/mol), <glycerin (−5.2 kcal/mol). Interestingly, 18 ligands (of 25 ligands) showed interaction with Gln 192 amino acid residue of human aldose reductase (HAR) as tabulated (Table S1; Figure 3a). The present finding was in good agreement with the previous report, where Gln 192 has been shown as one of the binding site residues of HAR (Kannayiram et al., 2022; Singh et al., 2017). On the other hand, five ligands (namely ligands 7, 8, 11, 17, and 19) do not exhibit any hydrogen bond interaction with HAR.

**TABLE 6:** The binding energy analysis of 25 selected (*Phoenix pusilla*) ligands with the human aldose reductase (HAR), protein tyrosine phosphatase 1B (PTP1B), human pancreatic alpha‐amylase (HPAA), peroxisome proliferator‐activated receptor gamma (HPPARG), and dipeptidyl peptidase IV (HDPP‐IV) by using the Auto Dock Vina method.

Ligand number	HAR	PTP1B	HPAA	HPPARG	HDPP‐IV
	Binding energy (kcal/mol)				
1	−5.7	−5.4	−4.0	−4.0	−4.7
2	−7.2	−4.5	−5.1	−4.5	−5.4
3	−6.0	−4.7	−4.3	−4.2	−5.0
4	−7.5	−4.2	−5.0	−4.7	−5.2
5	−5.2	−3.7	−4.0	−3.8	−4.5
6	−6.1	−4.3	−4.4	−4.0	−4.5
7	−7.8	−5.2	−5.4	−5.7	−5.4
8	−8.9	−7.1	−6.2	−6.1	−6.5
9	−6.8	−4.5	−4.7	−4.3	−5.0
10	−8.2	−6.0	−5.5	−5.4	−6.0
11	−7.6	−3.0	−4.8	−4.4	−4.8
12	−10.0	−5.3	−6.4	−6.1	−6.5
13	−8.0	−4.3	−4.9	−4.7	−5.1
14	−8.1	−5.4	−4.7	−4.7	−5.3
15	−8.4	−4.1	−6.0	−4.9	−5.7
16	−7.8	−6.3	−5.3	−4.9	−5.3
17	−8.0	−4.1	−5.0	−4.6	−4.8
18	−7.5	−5.3	−5.5	−4.6	−5.6
19	−8.1	−3.6	−4.6	−4.8	−5.1
20	−9.5	−7.2	−6.8	−6.4	−7.2
21	−7.7	−5.6	−5.2	−4.4	−5.4
22	−10.7	−7.6	−7.5	−6.4	−8.4
23	−8.0	−5.5	−4.9	−4.6	−5.8
24	−7.6	−5.5	−4.8	−4.4	−5.2
25	−8.2	−5.6	−5.3	−5.1	−5.8

Abbreviations: HAR, human aldose reductase; HDPP‐IV, human dipeptidyl peptidase IV; HPAA, human pancreatic alpha‐amylase; HPPARG, human peroxisome proliferator‐activated receptor gamma; PTP1B, human protein tyrosine phosphatase 1B.

The docking studies showed that 1‐formyl‐2,5‐dimethoxy‐6,9,10‐trimethyl‐anthracene had exhibited the maximum atomic contact energy (ACE) of −7.6 (kcal/mol) with human protein tyrosine phosphatase 1B (PTP1B) (Table 6). The binding energy results showed the following (highest to lowest) order: 1‐formyl‐2,5‐dimethoxy‐6,9,10‐trimethyl‐anthracene (−7.6 kcal/mol), <benzene propanoic acid, 3,5‐*bis*(1,1‐dimethylethyl)‐4‐hydroxy‐, methyl ester (−7.2 kcal/mol), <benzene, 1‐ethynyl‐4‐fluoro (−7.1 kcal/mol), <decanoic acid, 3‐methyl (−6.3 kcal/mol), <phenol, 2‐methoxy‐3‐(2‐propenyl) (−6.0 kcal/mol), <*n*‐hexadecanoic acid and 9,12‐octadecadienoic acid (Z,Z) (−5.6 kcal/mol), <9‐octadecenoic acid, methyl ester and methyl stearate (−5.5 kcal/mol), <6‐oxabicyclo[3.1.0] hexan‐2‐one and dodecanoic acid, methyl ester (−5.4 kcal/mol), <7‐epi‐*trans*‐sesquisabinene hydrate and 3‐deoxy‐d‐mannoic lactone (−5.3 kcal/mol), <tricyclo[2.2.1.0(2,6)] heptane, 1,3,3‐trimethyl (−5.2 kcal/mol), <β‐allyloxypropionic acid (−4.7 kcal/mol), <4H‐pyran‐4‐one, 2,3‐dihydro‐3,5‐dihydroxy‐6‐methyl and 5‐hydroxymethylfurfural (−4.5 kcal/mol), <dimethylamine, *N*‐(neopentyloxy) and Z‐10‐pentadecen‐1‐ol (−4.3 kcal/mol), <1‐butoxypropan‐2‐yl 2‐methylbutanoate (−4.2 kcal/mol), <trichloroacetic acid, 4‐tetradecyl ester and methyl tetradecanoate (−4.1 kcal/mol), <glycerin (−3.7 kcal/mol), <hexadecanoic acid, methyl ester (−3.6 kcal/mol), <cetene (−3.7 kcal/mol). Interestingly, 9 ligands (of 25 ligands) showed interaction with Phe 182 amino acid residue of human protein tyrosine phosphatase 1B (PTP1B) (Table S1; Figure 3b). The present finding was in good agreement with the previous report, where Phe 182 has been shown as one of the binding site residues of PTP1B (Liu et al., 2022; Rocha et al., 2021). On the other hand, 10 ligands (namely ligands 4, 6, 7, 8, 11, 15, 17, 19, 22, and 23) do not exhibit any hydrogen bond interaction with PTP1B.

The docking studies showed that 1‐formyl‐2,5‐dimethoxy‐6,9,10‐trimethyl‐anthracene had exhibited the maximum atomic contact energy (ACE) of −7.5 (kcal/mol) with HPAA as shown in Table 6. The binding energy results showed the following (highest to lowest) order: 1‐formyl‐2,5‐dimethoxy‐6,9,10‐trimethyl‐anthracene (−7.5 kcal/mol), <benzenepropanoic acid, 3,5‐*bis*(1,1‐dimethylethyl)‐4‐hydroxy, methyl ester (−6.8 kcal/mol), <7‐epi‐*trans*‐sesquisabinene hydrate (−6.4 kcal/mol), <benzene, 1‐ethynyl‐4‐fluoro (−6.2 kcal/mol), <trichloroacetic acid, 4‐tetradecyl ester (−6.0 kcal/mol), <phenol, 2‐methoxy‐3‐(2‐propenyl) and 3‐deoxy‐d‐mannoic lactone (−5.5 kcal/mol), <tricyclo[2.2.1.0(2,6)]heptane, 1,3,3‐trimethyl (−5.4 kcal/mol), <decanoic acid, 3‐methyl and 9,12‐octadecadienoic acid (Z,Z) (−5.3 kcal/mol), <*n*‐hexadecanoic acid (−5.2 kcal/mol), <4H‐pyran‐4‐one, 2,3‐dihydro‐3,5‐dihydroxy‐6‐methyl (−5.1 kcal/mol), <1‐butoxypropan‐2‐yl 2‐methylbutanoate and methyl tetradecanoate (−5.0 kcal/mol), <Z‐10‐pentadecen‐1‐ol and 9‐octadecenoic acid, methyl ester (−4.9 kcal/mol), <cetene and methyl stearate (−4.8 kcal/mol), <5‐hydroxymethylfurfural and dodecanoic acid, methyl ester (−4.7 kcal/mol), <hexadecanoic acid, methyl ester (−4.6 kcal/mol), <dimethylamine, *N*‐(neopentyloxy) (−4.4 kcal/mol), <β‐allyloxypropionic acid (−4.3 kcal/mol), <6‐oxabicyclo[3.1.0] hexan‐2‐one and glycerin (−4.0 kcal/mol). Interestingly, four ligands (namely ligands 13, 20, 21, and 22) showed interaction with Gln 63 amino acid residue of HPAA as tabulated (Table S1; Figure 3c).

The present finding was in good agreement with the previous report, where Gln 63 has been shown as one of the binding site residues of HPAA (Akshatha et al., 2021; Omar et al., 2022). On the other hand, nine ligands (namely ligands 4, 7, 8, 11, 14, 15, 17, 19, and 23) do not exhibit any hydrogen bond interaction with HPAA.

The docking studies showed that 1‐formyl‐2,5‐dimethoxy‐6,9,10‐trimethyl‐anthracene and benzene propanoic acid, 3,5‐*bis*(1,1‐dimethylethyl)‐4‐hydroxy‐, methyl ester have exhibited the maximum atomic contact energy (ACE) of −6.4 (kcal/mol) with human peroxisome proliferator–activated receptor gamma (HPPARG) (Table 6). The binding energy results showed the following (highest to lowest) order: 1‐formyl‐2,5‐dimethoxy‐6,9,10‐trimethyl‐anthracene and benzene propanoic acid, 3,5‐*bis*(1,1‐dimethylethyl)‐4‐hydroxy‐, methyl ester (−6.4 kcal/mol), <benzene, 1‐ethynyl‐4‐fluoro and 7‐epi‐*trans*‐sesquisabinene hydrate (−6.1 kcal/mol), <tricyclo[2.2.1.0(2,6)] heptane, 1,3,3‐trimethyl and decanoic acid, 3‐methyl (−5.4 kcal/mol), <9,12‐octadecadienoic acid (Z,Z) (−5.1 kcal/mol), <trichloroacetic acid, 4‐tetradecyl ester and decanoic acid, 3‐methyl (−4.9 kcal/mol), <hexadecanoic acid, methyl ester (−4.8 kcal/mol), <1‐butoxypropan‐2‐yl 2‐methylbutanoate, Z‐10‐pentadecen‐1‐ol and dodecanoic acid, methyl ester (−4.7 kcal/mol), <methyl tetradecanoate, 3‐deoxy‐d‐mannoic lactone and 9‐octadecenoic acid, methyl ester (−4.6 kcal/mol), <4H‐pyran‐4‐one, 2,3‐dihydro‐3,5‐dihydroxy‐6‐methyl (−4.5 kcal/mol), <cetene and methyl stearate (−4.4 kcal/mol), <5‐hydroxymethylfurfural (−4.3 kcal/mol), <β‐allyloxypropionic acid (−4.2 kcal/mol), <6‐oxabicyclo[3.1.0]hexan‐2‐one and dimethylamine, *N*‐(neopentyloxy) (−4.0 kcal/mol), <glycerin (−3.8 kcal/mol). Interestingly, two ligands (namely ligands 5 and 9) showed interaction with Ser 428 amino acid residue of HPPARG as tabulated (Table S1; Figure 3d). The present finding was in good agreement with the previous report, where Ser 428 has been shown as one of the binding site residues of HPPARG (Lakshmanan, 2020; Patchipala et al., 2022). On the other hand, 12 ligands do not exhibit any hydrogen bond interaction with HPPARG.

The docking studies showed that 1‐formyl‐2,5‐dimethoxy‐6,9,10‐trimethyl‐anthracene had exhibited the maximum atomic contact energy (ACE) of −8.4 (kcal/mol) with human dipeptidyl peptidase‐IV (HDPP‐IV) (Table 6). The binding energy results showed the following (highest to lowest) order: 1‐formyl‐2,5‐dimethoxy‐6,9,10‐trimethyl‐anthracene (−8.4 kcal/mol), <benzene propanoic acid, 3,5‐*bis*(1,1‐dimethylethyl)‐4‐hydroxy‐, methyl ester (−7.2 kcal/mol), <benzene, 1‐ethynyl‐4‐fluoro and 7‐epi‐*trans*‐sesquisabinene hydrate (−6.5 kcal/mol), <phenol, 2‐methoxy‐3‐(2‐propenyl) (−6.0 kcal/mol), <9‐octadecenoic acid, methyl ester and 9,12‐octadecadienoic acid (Z,Z) (−5.8 kcal/mol), <trichloroacetic acid, 4‐tetradecyl ester (−5.7 kcal/mol), <3‐deoxy‐d‐mannoic lactone (−5.6 kcal/mol), <4H‐pyran‐4‐one, 2,3‐dihydro‐3,5‐dihydroxy‐6‐methyl, tricyclo[2.2.1.0(2,6)] heptane, 1,3,3‐trimethyl and *n*‐hexadecanoic acid (−5.4 kcal/mol), <dodecanoic acid, methyl ester and decanoic acid, 3‐methyl (−5.3 kcal/mol), <1‐butoxypropan‐2‐yl 2‐methylbutanoate and methyl stearate (−5.2 kcal/mol), <Z‐10‐pentadecen‐1‐ol and hexadecanoic acid, methyl ester (−5.1 kcal/mol), <β‐allyloxypropionic acid and 5‐hydroxymethylfurfural (−5.0 kcal/mol), <cetene and methyl tetradecanoate (−4.8 kcal/mol), <6‐oxabicyclo[3.1.0]hexan‐2‐one (−4.7 kcal/mol), <glycerin and dimethylamine, *N*‐(neopentyloxy) (−4.5 kcal/mol). Interestingly, two ligands (ligands 1 and 18) showed interaction with His 740 amino acid residue of human dipeptidyl peptidase‐IV (HDPP‐IV) as tabulated (Table S1; Figure 3e). The present finding was in good agreement with the previous report, where His 740 has been shown as one of the binding site residues of HDPP‐IV (Liu et al., 2019). On the other hand, 11 ligands (namely ligands 4, 6, 7, 8, 11, 14, 15, 19, 20, 23, and 24) do not exhibit any hydrogen bond interaction with HDPP‐IV.

## 4 CONCLUSION

In this study, the methanolic extract of PPRF showed the IC_50_ as 72.60 and 69.86 μg/mL against PPAA and RIAG enzymes activities, respectively. Interestingly GC‐MS characterization showed the presence of 25 phytoconstituents from PPRF. Furthermore, all the 25 selected ligands of PPRF showed the potential to dock with all the five targeted enzymes namely (i) human aldose reductase (HAR), (ii) protein tyrosine phosphatase 1B (PTP1B), (iii) human pancreatic alpha‐amylase (HPAA), (iv) peroxisome proliferator‐activated receptor gamma (HPPARG), and (v) dipeptidyl peptidase IV (HDPP‐IV). Patch Dock study illustrates that 1‐formyl‐2,5‐dimethoxy‐6,9,10‐trimethyl‐anthracene (ligand number 22 of PPRF) exhibited the maximum atomic contact energy (ACE) for all the five target enzymes of DM. As an endnote, this research suggests that the methanolic extract of PPRF and its phytoconstituents can be considered as effective antidiabetic agents.

## AUTHOR CONTRIBUTIONS

**Kumaraswamy Srinivasan:** Conceptualization (equal); data curation (equal); formal analysis (equal); investigation (equal); methodology (equal); project administration (equal); software (equal); supervision (equal); validation (equal); visualization (equal); writing – original draft (equal). **Ammar B. Altemimi:** Conceptualization (equal); data curation (equal); formal analysis (equal); investigation (equal); methodology (equal); project administration (equal); resources (equal); software (equal); supervision (equal); validation (equal); writing – original draft (equal). **Radhakrishnan Narayanaswamy:** Conceptualization (equal); data curation (equal); formal analysis (equal); investigation (equal); methodology (equal); project administration (equal); resources (equal); software (equal); supervision (equal); validation (equal); writing – original draft (equal). **Prabhakaran Vasantha Srinivasan:** Conceptualization (equal); investigation (equal); methodology (equal); resources (equal); software (equal); visualization (equal); writing – original draft (equal); writing – review and editing (equal). **Mazin A. A. Najm:** Data curation (equal); formal analysis (equal); funding acquisition (lead); investigation (equal); methodology (equal); project administration (equal); visualization (equal); writing – original draft (equal); writing – review and editing (equal). **Nasser Mahna:** Data curation (equal); formal analysis (equal); funding acquisition (lead); investigation (equal); methodology (equal); project administration (equal); visualization (equal); writing – original draft (equal); writing – review and editing (equal).

## CONFLICT OF INTEREST STATEMENT

All authors declare there is no conflict of interest.

## Supporting information


Table S1

## Data Availability

The data that support the findings of this study are all available within the manuscript and Supporting Information. Additional data can be provided on request from the corresponding author.
