# Supplementary material for: GC‐MS, alpha‐amylase, and alpha‐glucosidase inhibition and molecular docking analysis of selected phytoconstituents of small wild date palm fruit (Phoenix pusilla)
Source: Food Sci Nutr. 2023 Jun 16;11(9):5304–17. doi: 10.1002/fsn3.3489 (PMC10494657; doi:10.1002/fsn3.3489)
Supplement: Supplementary file 1 — Table S1 [file FSN3-11-5304-s001.doc]

Supplementary Table 1 represents the interaction analysis of **twenty five selected (***Phoenix pusilla*) ligands with the human aldose reductase (HAR), protein tyrosine phosphatase 1B (PTP1B), pancreatic alpha-amylase (HPAA), peroxisome proliferator-activated receptor gamma (HPPARG) and dipeptidyl peptidase IV (HDPP-IV) by using the protein-ligand interaction profiler (PLIP) free web server.

| Ligand number | Human aldose reductase | | human protein tyrosine phosphatase 1B | | Human pancreatic alpha-amylase | | Human peroxisome proliferator-activated receptor gamma | | Human dipeptidyl peptidase IV | |
| --- | --- | --- | --- | --- | --- | --- | --- | --- | --- | --- |
|  | Amino acid | Bond distance (Å) | Amino acid | Bond distance (Å) | Amino acid | Bond distance (Å) | Amino acid | Bond distance (Å) | Amino acid | Bond distance (Å) |
| 1 | HB GLN 192  HB GLN 192  HB GLN 192  HB LYS 194 | HA 2.74 DA 3.23  HA 2.77 DA 3.72  HA 3.21 DA 3.99  HA 2.62 DA 3.04 | HB PHE 182  HB SER 216  HB ARG 221  HB ARG 221  HB ARG 221  HB GLN 266 | HA 2.39 DA 3.32  HA 2.86 DA 3.81  HA 2.42 DA 3.25  HA 1.95 DA 2.91  HA 2.70 DA 3.28  HA 2.09 DA 3.06 | HB LYS 457  HB HIS 491 | HA 2.16 DA 3.09  HA 2.15 DA 3.11 | HB HIS 323  HB GLU 324 | HA 2.26 DA 3.26  HA 2.43 DA 3.31 | HB TYR 631  HB HIS 740 | HA 2.31 DA 3.15  HA 2.87 DA 3.33 |
| 2 | HB THR 191  HB THR 191  HB GLN 192  HB GLN 192  HB GLN 192  HB GLN 192  HB GLU 193  HB ASN 292 | HA 3.18 DA 3.88  HA 3.35 DA 3.85  HA 2.34 DA 2.89  HA 2.05 DA 2.90  HA 2.31 DA 3.15  HA 2.38 DA 3.20  HA 3.19 DA 3.67  HA 2.66 DA 3.51 | HB ASN 139  HB ASN 162  HB THR 164  HB THR 164 | HA 3.17 DA 4.02  HA 2.32 DA 3.29  HA 2.64 DA 3.04  HA 2.16 DA 3.04 | HB GLY 304  HB GLY 309  HB ARG 346 | HA 2.96 DA 3.32  HA 3.23 DA 4.01  HA 2.47 DA 3.34 | HB ILE 281 | HA 2.35 DA 2.83 | HB THR 350  HB THR 351  HB SER 376 | HA 2.34 DA 3.25  HA 2.17 DA 3.00  HA 2.43 DA 2.99 |
| 3 | HB THR 191  HB GLN 192  HB GLN 192  HB GLN 192  HB GLN 192  HB GLN 192  HB ARG 296  HB ARG 296  HB ARG 296  HB ARG 296 | HA 3.33 DA 3.78  HA 2.42 DA 3.24  HA 2.28 DA 3.36  HA 3.44 DA 3.84  HA 3.26 DA 4.02  HA 2.46 DA 3.04  HA 1.88 DA 2.84  HA 2.09 DA 2.97  HA 2.40 DA 3.16  HA 2.64 DA 3.20 | HB LYS 120  HB PHE 182  HB ARG 221  HB ARG 221  HB GLN 266 | HA 2.15 DA 2.99  HA 2.31 DA 3.25  HA 3.44 DA 3.93  HA 3.02 DA 4.01  HA 2.08 DA 3.03 | HB ARG 398  HB ARG 421  HB ARG 421 | HA 2.60 DA 3.19  HA 2.26 DA 3.16  HA 2.41 DA 3.28 | - | - | HB GLU 347  HB SER 349  HB THR 350  HB THR 351  HB GLY 355 | HA 2.83 DA 3.76  HA 3.53 DA 3.88  HA 1.99 DA 2.80  HA 2.12 DA 2.96  HA 1.86 DA 2.84 |
| 4 | HB GLN 192  HB GLN 192  HB ARG 296  HB ARG 296 | HA 2.70 DA 3.15  HA 2.59 DA 3.55  HA 2.54 DA 3.14  HA 2.56 DA 3.26 | - | - | - | - | - | - | - | - |
| 5 | HB GLN 192  HB GLN 192  HB ARG 296  HB ARG 296  HB ARG 296  HB ARG 296  HB ARG 296  HB ARG 296 | HA 2.35 DA 3.20  HA 2.20 DA 3.33  HA 2.11 DA 3.02  HA 2.28 DA 3.10  HA 2.46 DA 3.13  HA 2.43 DA 3.02  HA 2.22 DA 3.11  HA 2.20 DA 3.02 | HB PHE 182  HB ARG 221  HB ARG 221  HB ARG 221  HB GLN 266 | HA 2.31 DA 3.24  HA 2.13 DA 3.04  HA 2.29 DA 3.14  HA 2.73 DA 3.31  HA 2.05 DA 3.01 | HB ARG 267  HB GLN 302  HB ILE 312  HB THR 314  HB THR 314  HB ARG 346 | HA 3.03 DA 3.60  HA 2.42 DA 3.11  HA 2.16 DA 2.91  HA 2.52 DA 3.19  HA 2.93 DA 3.37  HA 2.64 DA 3.10 | HB HIS 425  HB SER 428  HB PHE 432 | HA 2.20 DA 2.99  HA 2.42 DA 3.12 HA 2.03 DA 3.03 | HB GLN 320  HB GLY 352  HB ARG 669  HB GLY 672 | HA 2.40 DA 3.19  HA 2.44 DA 3.28  HA 2.26 DA 2.88  HA 2.40 DA 3.03 |
| 6 | HB GLN 192  HB ARG 296  HB ARG 296 | HA 3.49 DA 4.00  HA 2.27 DA 2.91  HA 2.20 DA 2.96 | - | - | HB ARG 398  HB ARG 398  HB ASP 402 | HA 2.73 DA 3.16  HA 2.90 DA 3.30  HA 3.18 DA 3.72 | HB ARG 212  HB SER 382  HB GLN 420 | HA 2.96 DA 3.92  HA 2.39 DA 3.25  HA 2.10 DA 3.09 | - | - |
| 7 | - | - | - | - | - | - | - | - | - | - |
| 8 | - | - | - | - | - | - | - | - | - | - |
| 9 | HB GLN 192  HB LYS 194  HB LYS 194  HB LEU 195  HB ARG 296  HB ARG 296  HB ARG 296  HB ARG 296 | HA 2.38 DA 3.02  HA 3.18 DA 3.55  HA 2.62 DA 3.55  HA 2.58 DA 3.55  HA 2.98 DA 2.99  HA 3.13 DA 3.64  HA 2.10 DA 2.97  HA 2.47 DA 3.23 | HB SER 203 | HA 3.13 DA 3.94 | HB ARG 195  HB ARG 195  HB ASP 197  HB HIS 299  HB ASP 300  HB HIS 305 | HA 3.41 DA 4.08  HA 2.08 DA 3.06  HA 2.20 DA 3.07  HA 3.39 DA 4.06  HA 3.21 DA 3.62  HA 2.76 DA 3.17 | HB PRO 426  HB SER 428 | HA 2.15 DA 2.83  HA 2.46 DA 3.18 | HB TYR 662 | HA 2.38 DA 2.76 |
| 10 | HB GLN 192  HB LEU 195  HB ARG 296  HB ARG 296 | HA 3.27 DA 3.88  HA 3.80 DA 4.10  HA 2.63 DA 3.20  HA 2.61 DA 3.29 | HB GLU 115  HB LYS 120  HB SER 216  HB ARG 221 | HA 3.29 DA 3.81  HA 2.10 DA 3.03  HA 3.68 DA 4.04  HA 2.51 DA 3.01 | HB HIS 299  HB ASP 300 | HA 2.52 DA 3.40  HA 2.64 DA 3.23 | HB ASN 335 | HA 2.07 DA 2.80 | HB LYS 71  HB ASN 74 | HA 2.81 DA 3.27  HA 2.19 DA 2.92 |
| 11 | - | - | - | - | - | - | - | - | - | - |
| 12 | HB GLN 192  HB GLN 192  HB ARG 296  HB ARG 296  HB ARG 296  HB ARG 296 | HA 2.95 DA 3.58  HA 2.85 DA 3.64  HA 3.30 DA 3.85  HA 3.57 DA 3.97  HA 1.81 DA 2.79  HA 1.93 DA 2.91 | HB THR 138 | HA 3.63 DA 3.93 | HB GLU 233 | HA 2.03 DA 2.87 | HB GLU 471 | HA 2.48 DA 3.14 | HB TYR 381  HB THR 401 | HA 3.11 DA 3.86  HA 1.95 DA 2.80 |
| 13 | HB GLN 192  HB ARG 296  HB ARG 296  HB ARG 296 | HA 2.59 DA 3.11  HA 2.59 DA 3.02  HA 2.26 DA 2.86  HA 2.37 DA 3.02 | HB ARG 45  HB LEU 119  HB SER 216 | HA 2.18 DA 3.11  HA 2.61 DA 3.21  HA 3.74 DA 4.08 | HB TRP 59  HB GLN 63 | HA 2.23 DA 3.00  HA 2.43 DA 2.98 | HB ARG 280 | HA 2.33 DA 3.07 | HB TRP 629  HB TRP 629 | HA 1.91 DA 2.92  HA 2.59 DA 2.97 |
| 14 | HB GLN 192  HB GLN 192 | HA 2.29 DA 3.12  HA 2.14 DA 3.24 | HB PHE 182  HB ARG 221  HB GLN 266 | HA 2.31 DA 3.29  HA 2.55 DA 3.20  HA 2.33 DA 3.26 | - | - | - | - | - | - |
| 15 | HB GLN 192  HB LYS 194  HB LEU 195 | HA 3.20 DA 3.65  HA 3.15 DA 3.33  HA 3.12 DA 3.60 | - | - | - | - | - | - |  |  |
| 16 | HB GLN 192  HB GLN 192 | HA 2.29 DA 3.08  HA 2.16 DA 3.18 | HB PHE 182  HB ARG 221  HB GLN 266 | HA 2.43 DA 3.32  HA 2.22 DA 3.20  HA 1.92 DA 2.89 | HB ASP 300 | HA 2.21 DA 3.12 | HB LEU 330 | HA 3.50 DA 3.86 | HB ASN 92  HB PHE 95 | HA 1.87 DA 2.84  HA 2.11 DA 3.03 |
| 17 | - | - | - | - | - | - | - | - | HB ILE 102 | HA 2.28 DA 3.09 |
| 18 | HB HIS 163  HB HIS 163  HB GLN 192  HB GLN 192  HB LEU 195  HB ARG 296  HB ARG 296 | HA 2.53 DA 3.15  HA 2.26 DA 3.31  HA 2.03 DA 2.87  HA 2.55 DA 3.11  HA 3.30 DA 3.63  HA 2.70 DA 3.10  HA 2.35 DA 2.92 | HB PHE 182  HB ALA 217  HB ARG 221  HB ARG 221 | HA 2.46 DA 3.41  HA 3.30 DA 3.80  HA 2.34 DA 2.98  HA 2.75 DA 3.30 | HB ARG 267  HB ARG 267  HB THR 314  HB ASP 317 | HA 2.33 DA 3.08  HA 2.97 DA 3.59  HA 3.46 DA 3.88  HA 2.38 DA 3.10 | HB LYS 261 | HA 2.35 DA 3.04 | HB ASN 710  HB ASP 739  HB HIS 740  HB HIS 740  HB GLY 741 | HA 1.86 DA 2.72  HA 2.70 DA 3.25  HA 2.98 DA 3.80  HA 2.29 DA 3.29  HA 2.27 DA 2.92 |
| 19 | - | - | - | - | - | - | - | - | - | - |
| 20 | HB THR 191  HB GLN 192  HB GLN 192  HB GLU 193  HB ASN 292 | HA 3.35 DA 3.97  HA 2.24 DA 3.05  HA 2.66 DA 3.19  HA 3.82 DA 4.07  HA 3.18 DA 3.75 | HB SER 216  HB ALA 217 | HA 2.88 DA 3.63  HA 2.04 DA 3.04 | HB TRP 59  HB GLN 63 | HA 2.24 DA 2.94  HA 2.37 DA 3.15 | - | - | - | - |
| 21 | HB THR 191  HB THR 191  HB THR 191  HB GLN 192  HB GLN 192  HB GLU 193  HB ASN 292  HB ASN 294 | HA 2.52 DA 3.09  HA 2.56 DA 3.26  HA 2.70 DA 3.27  HA 2.13 DA 2.94  HA 2.62 DA 3.14  HA 3.28 DA 3.69  HA 2.69 DA 3.08  HA 2.58 DA 3.28 | HB PHE 182  HB GLY 220  HB ARG 221  HB GLN 266 | HA 2.40 DA 3.30  HA 3.47 DA 3.93  HA 2.00 DA 3.00  HA 1.94 DA 2.91 | HB GLN 63 | HA 2.84 DA 3.32 | HB GLN 314 | HA 2.13 DA 2.91 | HB SER 630  HB TYR 662  HB ASN 710 | HA 2.73 DA 3.30  HA 2.84 DA 3.20  HA 2.41 DA 3.20 |
| 22 | HB HIS 163  HB HIS 163  HB HIS 163  HB LEU 164  HB LEU 164 | HA 2.87 DA 3.56  HA 2.94 DA 3.64  HA 2.21 DA 2.95  HA 2.97 DA 3.77  HA 2.80 DA 3.98 | - | - | HB GLN 63 | HA 2.43 DA 2.98 | - | - | HB LYS 71  HB ILE 102 | HA 2.18 DA 2.88  HA 2.68 DA 3.58 |
| 23 | HB GLU 193 | HA 2.76 DA 3.15 | - | - | - | - |  | - | - | - |
| 24 | HB GLN 192 | HA 2.85 DA 3.37 | HB PHE 182 | HA 1.94 DA 2.94 | HB THR 163 | HA 2.44 DA 2.83 | - | - | - | - |
| 25 | HB THR 191  HB THR 191  HB THR 191  HB GLN 192  HB GLN 192  HB GLN 192  HB ASN 292 | HA 2.73 DA 3.38  HA 2.86 DA 3.40  HA 2.81 DA 3.59  HA 3.33 DA 3.99  HA 2.30 DA 3.07  HA 2.42 DA 3.31  HA 2.74 DA 3.28 | HB PHE 182  HB ARG 221  HB GLN 266 | HA 2.33 DA 3.27  HA 2.65 DA 3.21  HA 1.98 DA 2.93 | HB ALA 198 | HA 3.58 DA 3.94 | HB GLU 272 | HA 3.04 DA 3.78 | HB ARG 356  HB PHE 357  HB ARG 358 | HA 2.56 DA 3.13  HA 1.80 DA 2.81  HA 2.16 DA 3.05 |
